# Blood RNA-Seq profiling reveals a set of circular RNAs differentially expressed in frail individuals

**DOI:** 10.1186/s12979-023-00356-6

**Published:** 2023-07-11

**Authors:** Leire Iparraguirre, Ainhoa Alberro, Saioa GS Iñiguez, Maider Muñoz-Culla, Itziar Vergara, Ander Matheu, David Otaegui

**Affiliations:** 1 Multiple Sclerosis Unit. Biodonostia Health Research Institute, 20014 San Sebastián, Spain; 2 CIBERNED, MADRID, Spain; 3 Department of Basic Psychological Processes and Their Development, Euskal Herriko Unibertsitatea (UPV/EHU), 20018, San Sebastián, Spain; 4 Group of Research in Primary Care. Biodonostia Health Research Institute, 20014 San Sebastián, Spain; 5 Research Network on Chronicity, Primary Care and Health Promotion (RICAPPS), San Sebastián, Spain; 6 Cellular oncology group. Biodonostia Health Research Institute, 20014 San Sebastián, Spain; 7 IKERBASQUE, Basque Foundation for Science, Bilbao, Spain; 8 Centro de Investigación Biomédica en Red de Fragilidad y Envejecimiento (CIBERfes), Carlos III Institute, Madrid, Spain

**Keywords:** Frailty, Circular RNA, Biomarker

## Abstract

**Background:**

Frailty is an intermediate and reversible geriatric syndrome that often precedes dependence. Therefore, its identification is essential to prevent dependence. Several molecules have been proposed as biomarkers of frailty, but none of them have reached clinical practice. Recently, circular RNAs have emerged as new non-coding RNAs. Their regulatory role together with their high stability in biofluids makes them good candidates as biomarkers for various processes, but, to date, no study has characterized the expression of circRNA in frailty.

**Results:**

We studied RNA from leukocytes of 35 frails and 35 robust individuals. After RNA-Sequencing, circRNA detection was performed by CIRI2 and Circexplorer2 and differential expression analysis by DESeq2. Validation was performed by Quantitative-PCR. Linear Discriminant Analysis was performed to determine the best circRNA combination to discriminate frail from robust. In addition, CircRNA candidates were studied in 13 additional elder donors before and after a 3-month physical intervention. We found 89 differentially expressed circRNAs (*p*-value<0.05, FC>|1.5|) with frailty. Upregulation of hsa_circ_0007817, hsa_circ_0101802 and hsa_circ_0060527 in frail individuals was validated. The combination of hsa_circ_0079284, hsa_circ_0007817 and hsa_circ_0075737 levels showed a great biomarker value with a 95.9% probability of correctly classifying frail and robust individuals. Moreover, hsa_circ_0079284 levels decreased after physical intervention in concordance with an improvement in frailty scores.

**Conclusions:**

This work describes for the first time a different expression pattern of circular RNA (circRNAs) between frail and robust individuals. Moreover, the level of some circRNAs is modulated after a physical intervention. These results suggest that they could be used as minimally invasive biomarkers of frailty.

**Supplementary Information:**

The online version contains supplementary material available at 10.1186/s12979-023-00356-6.

## Background

Life expectancy has notably increased in the last decades. However, healthy life expectancy has not increased that much and, therefore, there is a growing period of morbidity [17]. Interestingly, disability and dependency in older adults are commonly preceded by frailty. Indeed, frailty is the main risk factor for the development of disability [41]. For this reason, the detection of frailty is the first step to being able to intervene and, potentially, prevent or delay dependency.

Frailty is characterized by a reduced functional reserve, impaired adaptive capacity across multiple physiological systems, and increased vulnerability to stressors [6]. In contrast, an older adult is classified as robust when the functional capacity is conserved, and phenotypic stability (or performance) is conserved after the occurrence of a clinical stressor [37]. Frailty is a heterogeneous state comprising physical, psychological and cognitive impairment, and even if it has been widely studied, there is no consensus on an operational definition of frailty and on the best tools to identify it [34]. It is generally accepted that frailty is a reversible state. Indeed, physical and nutritional interventions have been proposed to recover robustness [24].

There are numerous tests based on clinical and functional measures to identify frailty. Each test evaluates different aspects of frailty and, therefore, the prevalence of frailty varies widely depending on the test applied [4]. The classification proposed by Fried and collaborators [13] and the Canadian Study of Health and Aging Frailty Index [32] are probably the most used scales in research. However, they are complex and time-consuming, and many geriatricians and clinicians consider that they cannot be applied in everyday clinics. Therefore, several works have been carried out to find easier and faster tools to evaluate frailty. These include the use of a single functional measure, such as the Timed up-and-go [30] or Gait Speed [33]. Similarly, based on the number of citations, the Short Physical Performance Battery (SPPB) which was published already in 1994 by Guralnik and colleagues [19], is gaining importance in the last years for the identification of frailty.

Parallel to studying and comparing tests and scales, research on biomarkers of frailty is being conducted to complement them and to better understand the biology of frailty [11]. Inflammatory, endocrine, metabolic, genet ic, and epigenetic biomarkers have been proposed, with controversial results and, therefore, they are not used in clinical practice [2, 3, 5]. For this reason, we should continue investigating new potential biomarkers that could help the identification of frailty. Several works have demonstrated that the transcriptome and its regulations are clearly affected by age, and some of these works also found changes in the blood transcriptome with frailty [38], however, none of them studied the circular RNAs.

Circular RNAs (circRNAs) have emerged in the last years as a new type of endogenous non-coding transcripts playing an important role in several biological processes [27]. They are characterized by their covalently closed structure formed by an alternative splicing process called backsplicing, by which a downstream splice donor and an upstream splice acceptor are joined together creating a circRNA-exclusive junction called back-splice junction (BSJ). Their circular structure endows them with high stability which, along with their presence in different biofluids and their involvement in different physiological and pathological processes [25], has attracted the interest of many researchers as a promising source of biomarkers. Importantly, among the different physiological and pathological processes, circRNAs have been linked to aging [22], longevity [16], and age-associated phenotypes such as immunosenescence [40] and mitochondrial dysfunction [36]. Nevertheless, to our knowledge, so far, circRNAs have not been studied in relation to frailty.

In this study, we have profiled by RNA-Seq for the first time the circRNAs from the blood of elderly frail and robust individuals including the analysis of their biomarker potential. Additionally, we also present a preliminary study of circRNA expression upon a physical intervention in elderly individuals.

## Results

### The circular transcriptome significantly differs between frail and robust individuals

**Fig. 1:**
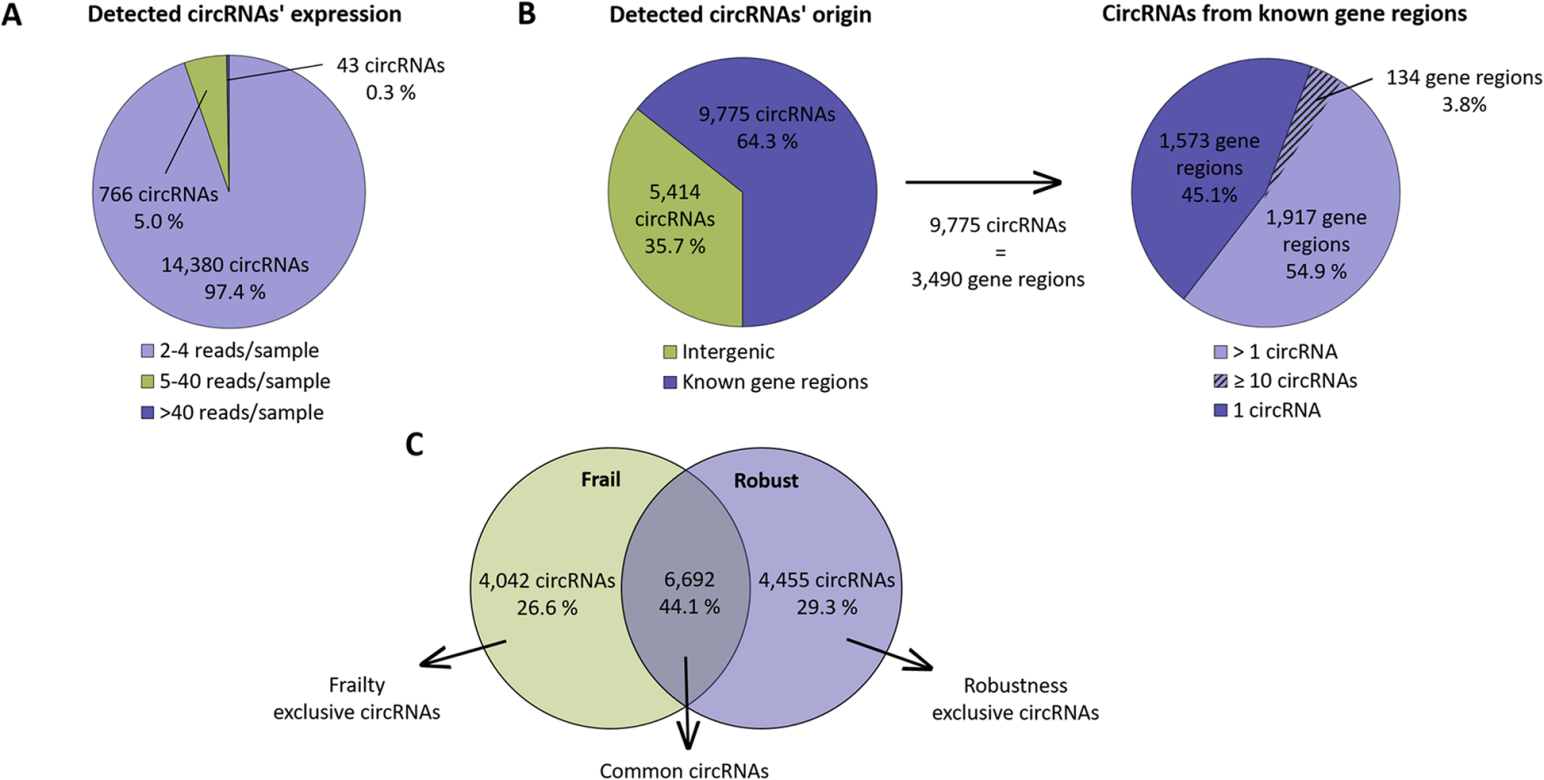
General description of the circRNAs detected by RNA-Seq. CircRNAs have been depicted based on their base mean (**A**) and genomic origin (**B**). **C** Number of circRNAs detected either exclusively in one of the groups or in both

For the RNAseq, samples were pooled (see details at the methods section), and 20 pools were sequenced. CircExplorer2 and CIRI2 detected a total of 52,835 and 122,727 circRNAs, respectively in all the samples. A good detection overlap was observed between algorithms with 41,108 circRNAs in common, out of which 15,189 were detected with a minimum of 2 reads and, therefore, were considered *bona fide* circRNAs. Most of those circRNAs are poorly expressed, with 94.7% detected with less than 5 reads/sample (14,380 circRNAs). Of the highly expressed circRNAs, 766 (5.0%) are detected with 5-40 reads and only a few (43 circRNAs, 0.3%) are detected with more than 40 reads (Fig. 1A and Supplementary Fig. 1A).

When the origin of identified circRNAs was investigated, we found that 64.3% of the circRNAs (9,775 circRNAs) are located in a known gene locus, while 5,414 are intergenic. Remarkably, although we have detected 9,775 genic circRNAs, only 3,490 unique genes are identified, indicating that some of the circRNAs originated from the same genic region. Indeed, out of the 3,490 genic regions, 1,917 regions produce more than one circRNA, and 134 regions could be considered circRNA hotspots for producing 10 or more circRNAs (Fig. 1B and Supplementary Fig. 1B). Regarding the comparison between robust and frail individuals, out of the 15,189 circRNAs, 44.1% (6,692 circRNAs) are detected both in frail and robust participants while the rest are exclusive to one of the groups. There are 4,042 circRNAs detected in at least one of the pools from frail individuals and absent in all the robust ones. Similarly, we detected 4,455 circRNAs which are exclusively detected in some of the pools corresponding to robust individuals (Fig. 1C).

**Fig. 2:**
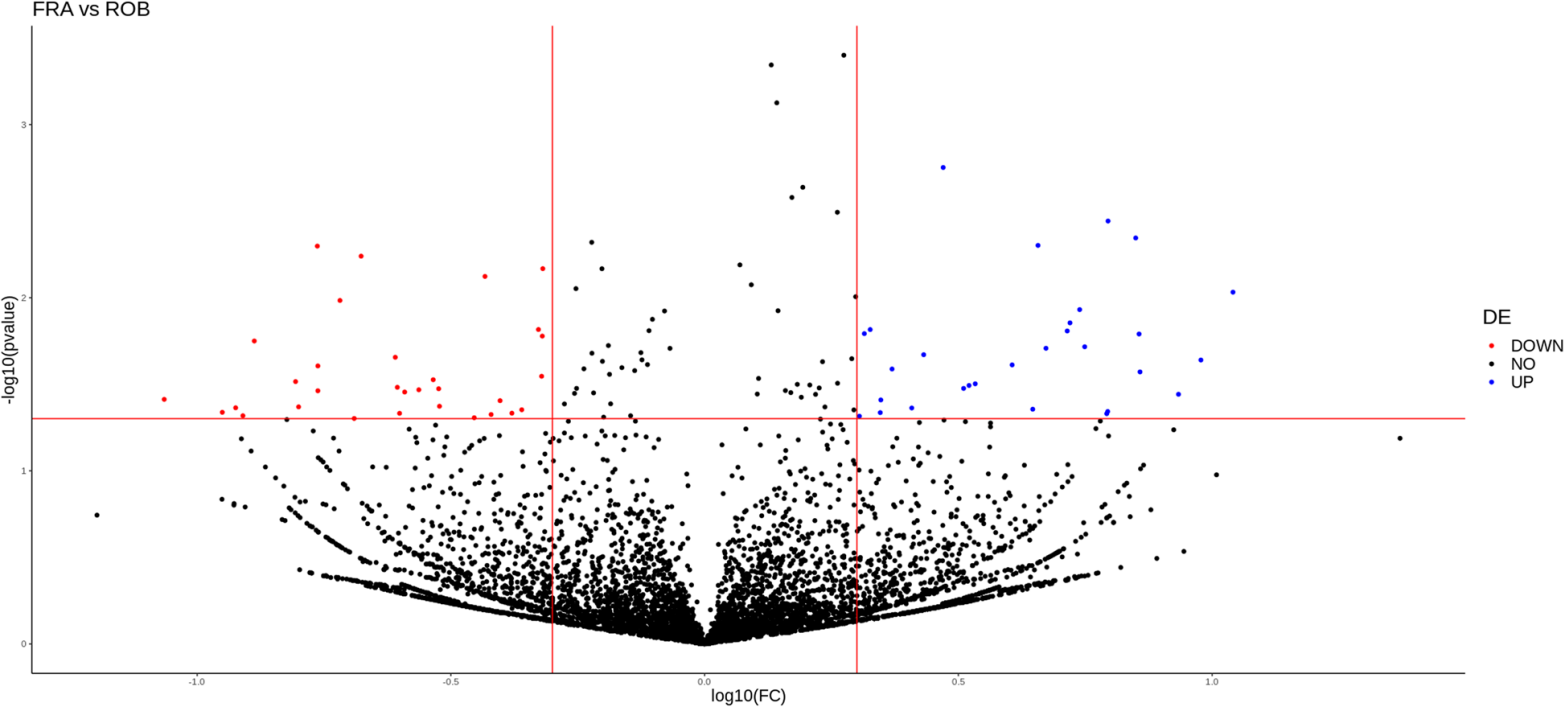
DE circRNAs between frail and robust individuals. Volcano plots displaying the differential expression analysis results for circRNAs between frail and robust individuals. The 5 circRNAs selected for qPCR validation are indicated. Abbreviations: FC, fold change

For the circRNAs detected in both groups, we performed a differential expression analysis to report the expression differences that could be associated with frailty. Interestingly, we identified 89 significantly differentially expressed (DE) circRNAs, with a similar proportion of upregulated and downregulated circRNAs - 43 circRNAs were found to be upregulated in frail individuals whereas 46 were downregulated (Fig. 2 and Supplementary Table 1).

In order to select circRNA candidates for their subsequent validation by qPCR, we included some additional criteria. Among the circRNAs found to be DE we filtered out those detected with a low number of reads (Base Mean < 5 reads/sample) and selected the top 5 differentially expressed circRNAs based on their Fold Change (FC > |1.8|). Additionally, among the group exclusive circRNAs, the two circRNAs with the highest Base Mean were selected for validation.

**Fig. 3:**
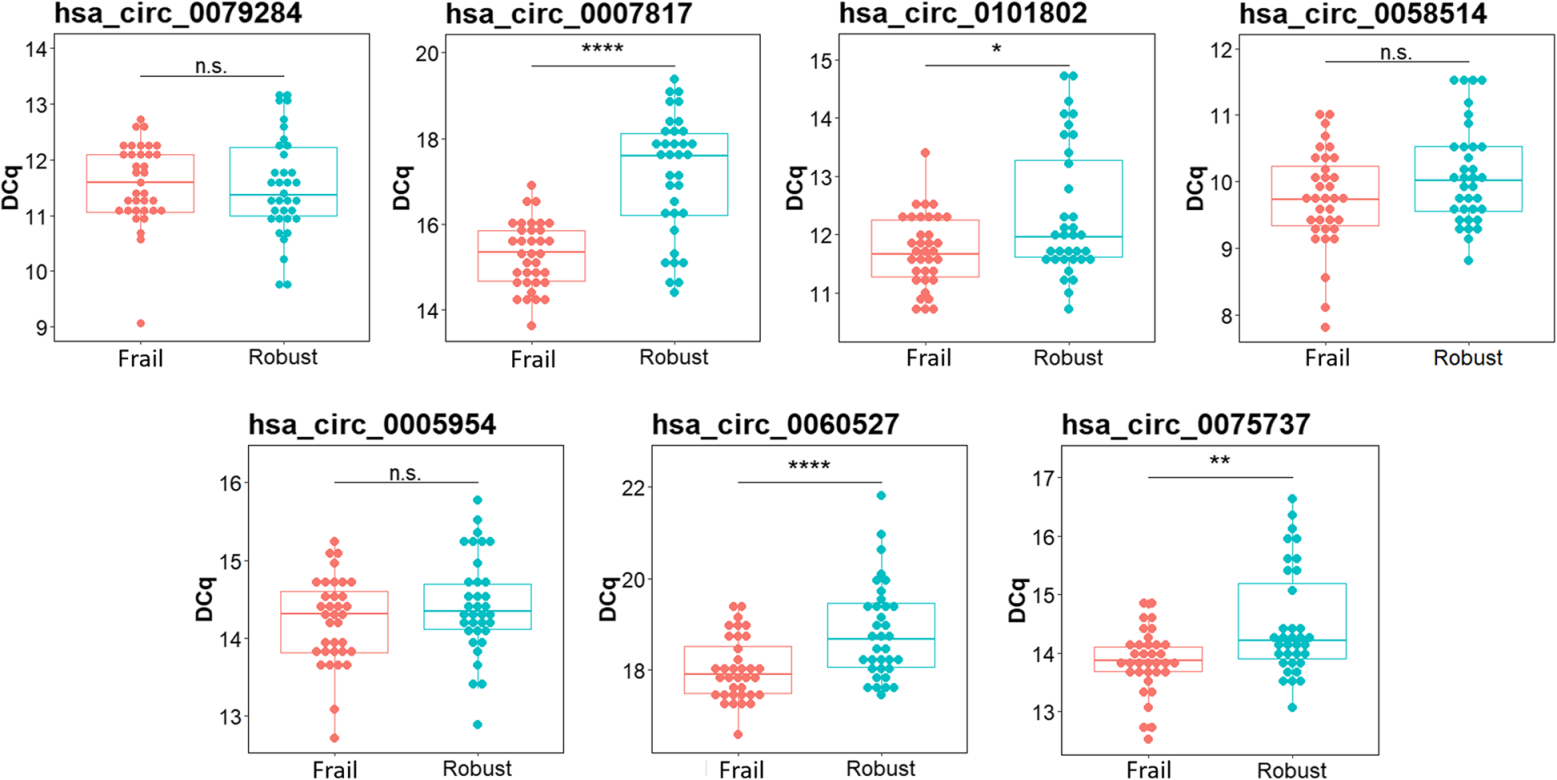
Four of the circRNA candidates are found to be upregulated in frail individuals assessed by qPCR. All the 35 frail and 35 robust individuals were studied separately during this validation. T-test or Wilcoxon rank sum tests were performed for the statistical analysis of normally and non-normally distributed variables, respectively. Note that a higher DCq value corresponds to a lower expression of the circRNA. Abbreviations and symbols: n.s., not significant; *, *p*-value ≤ 0.05;**, *p*-value ≤ 0.01; ****, *p*-value ≤ 0.0001

The validation was performed on all 70 individuals enrolled. Samples were studied independently, without pooling them. qPCRs were conducted using divergent primers spanning the respective BSJ for each of the 7 circRNA candidates. The correct amplification of the BSJs was confirmed by agarose gel electrophoresis and Sanger sequencing (Supplementary Fig. 2). 4 of the circRNAs selected from the RNA-Seq analysis were found significantly upregulated by qPCR. The circRNA hsa_circ_0007817 was found to be 2.11 times more expressed in frails when compared to robust individuals (*p*<0.0001) and for hsa_circ_0101802 we validated an upregulation of 1.24 times (*p*-value=0.03). Remarkably, the two circRNAs selected for being group exclusive could be detected in all the 70 individuals, indicating that their presence/absence cannot be used as a clear criterion for fail/robust classification. Hsa_circ_0060527 and hsa_circ_0075737, were significantly upregulated in frail individuals (FC=1.39; *p*-value<0.0001 and FC=1.26; *p*-value=0.005, respectively) (Fig. 3 and Table 1).

**Table 1:** Main clinical and demographic characteristics of the individuals enrolled in the study

	**Discovery and validation cohort**		**Intervention cohort**		
	**Robust** (*N*=35)	**Frail** (*N*=35)	**Pre** (*N*=13)	**Post** (*N*=12)	**Post + 3m** (*N*=6)
**Sex**					
Female	20	20	8	8	4
Male	15	15	5	4	2
**Age** **(years)**					
Inclusion criteria	>70 years		>70 years		
Mean (SD)	78.8 (±2.63)	81.0 (±4.96)	78.2(±3.34)	78.4(±3.48)	77.1(±4.27)
**Timed up-and-go (TUG)** (seconds)					
Inclusion criteria	<12 s	≥12 s			
Mean (SD)	8.48 (±0.88)	15.7 (±3.05)	10.5(±2.17)	9.8(±1.41)	9.8(±1.15)
**Gait Speed (GA) **(m/s)					
Inclusion criteria	>0.8	≤0.8			
Mean (SD)	1.13 (±0.12)	0.64 (±0.10)	-	-	-
**Short Physical Performance Battery (SPPB) **(points)					
Inclusion criteria	≥10	<10			
Mean (SD)	11.1 (±0.69)	7.2 (±1.21)	7.2 (±1.74)	8.3 (±1.67)	7.0 (±1.55)

We also evaluated whether the expression of the 7 candidate circRNAs correlates with the loss of functional abilities. The obtained results show that the expression of hsa_circ_0007817, hsa_circ_0101802, hsa_circ_0058514, hsa_circ_0060527, and hsa_circ_0075737 gradually increase with the time required by the elderly to complete the TUG test. In line with this, as the GS score decreases, the levels of hsa_circ_0007817, hsa_circ_0101802, hsa_circ_0060527 and hsa_circ_0075737 increase. Similarly, the levels of hsa_circ_0007817 and hsa_circ_0060527 increase as the SPPB score decreases. Therefore, a higher expression of the mentioned circRNAs is associated with a worse score in the frailty tests (Supplementary Table 2).

### circRNAs are potential biomarkers of frailty

**Fig. 4:**
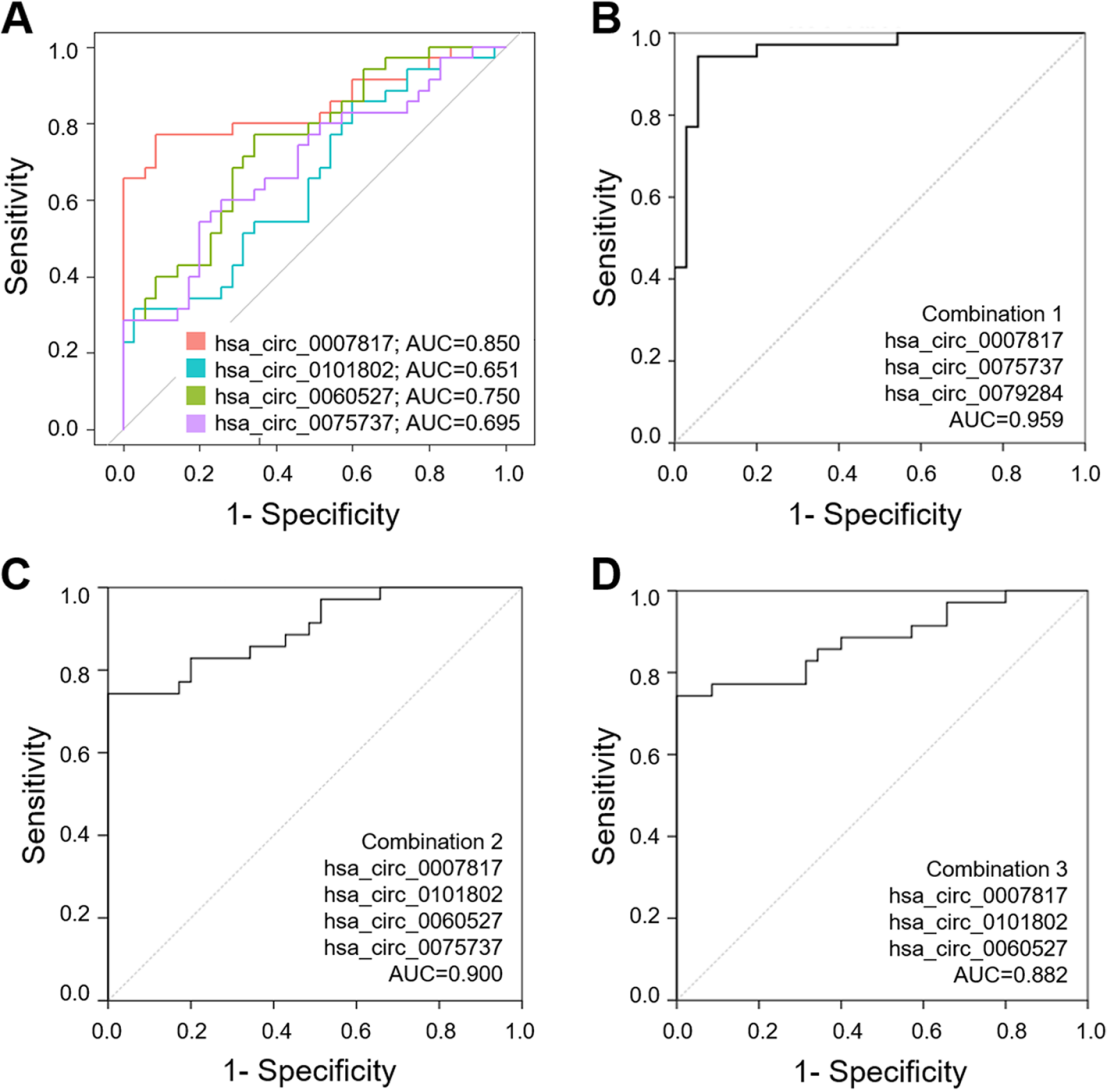
ROC curves showing the potential circRNAs to be biomarkers in frailty. **A** A ROC curve for each of the four validated circRNAs is represented. **B**, **C** and **D** plots represent the ROC curves obtained by combining different circRNAs. Their corresponding AUC values for each of the curves are shown in the lower right corner

To explore the diagnostic potential of the aforementioned circRNA candidates, we performed a ROC curve analysis. As shown in Fig. 4, the 4 validated circRNAs showed a good performance with an area under the curve (AUC) higher than 0.65, indicating that based on the leukocyte expression of any of those circRNAs the probability of correctly classifying them as frail or robust is higher than 65%. Remarkably, the AUC calculated for hsa_circ_0007817 is 0.85 which shows a great potential to function as a frailty biomarker.

Additionally, we investigated whether the use of several circRNAs in combination could improve the potential to correctly discriminate between frails and robust. To do so, we created a Linear Discriminant Analysis to detect the best variables or combination of variables that better discriminate the frailty status. Based on this model, we reported that the combination of the expression levels for hsa_circ_0007817, hsa_circ_0075737 and hsa_circ_0079284 (Combination 1) performs with an AUC of 0.959, indicating that it has a great diagnostic value, with a 95.9% probability of correctly classifying the frail and robust individuals (Fig. 4). Moreover, the combinations of the four significant and validated circRNAs (Combination 2: hsa_circ_0007817, hsa_circ_0101802, hsa_circ_0060527, hsa_circ_0075737) and the combination of hsa_circ_0007817 + hsa_circ_0101802 + hsa_circ_0060527 (Combination 3) also outperform the discriminating capacity of individual circRNAs (AUC 0.900 and 0.882, respectively) (Fig. 4 and Supplementary Table 3).

### The expression of hsa_circ_0079284 is reduced after a physical intervention

Then, we investigated circRNA expression in elder donors after a physical intervention. The expression of the five circRNAs with more promising results regarding their potential to be frailty biomarkers (hsa_circ_0007817, hsa_circ_0101802, hsa_circ_0060527, hsa_circ_0075737, and hsa_circ_0079284) was determined in these samples by qPCR. Only three out of the five selected circRNAs were sufficiently amplified in these samples. No consistent data was obtained for hsa_circ_0007817 and hsa_circ_0060527 due to their low expression.

**Fig. 5:**
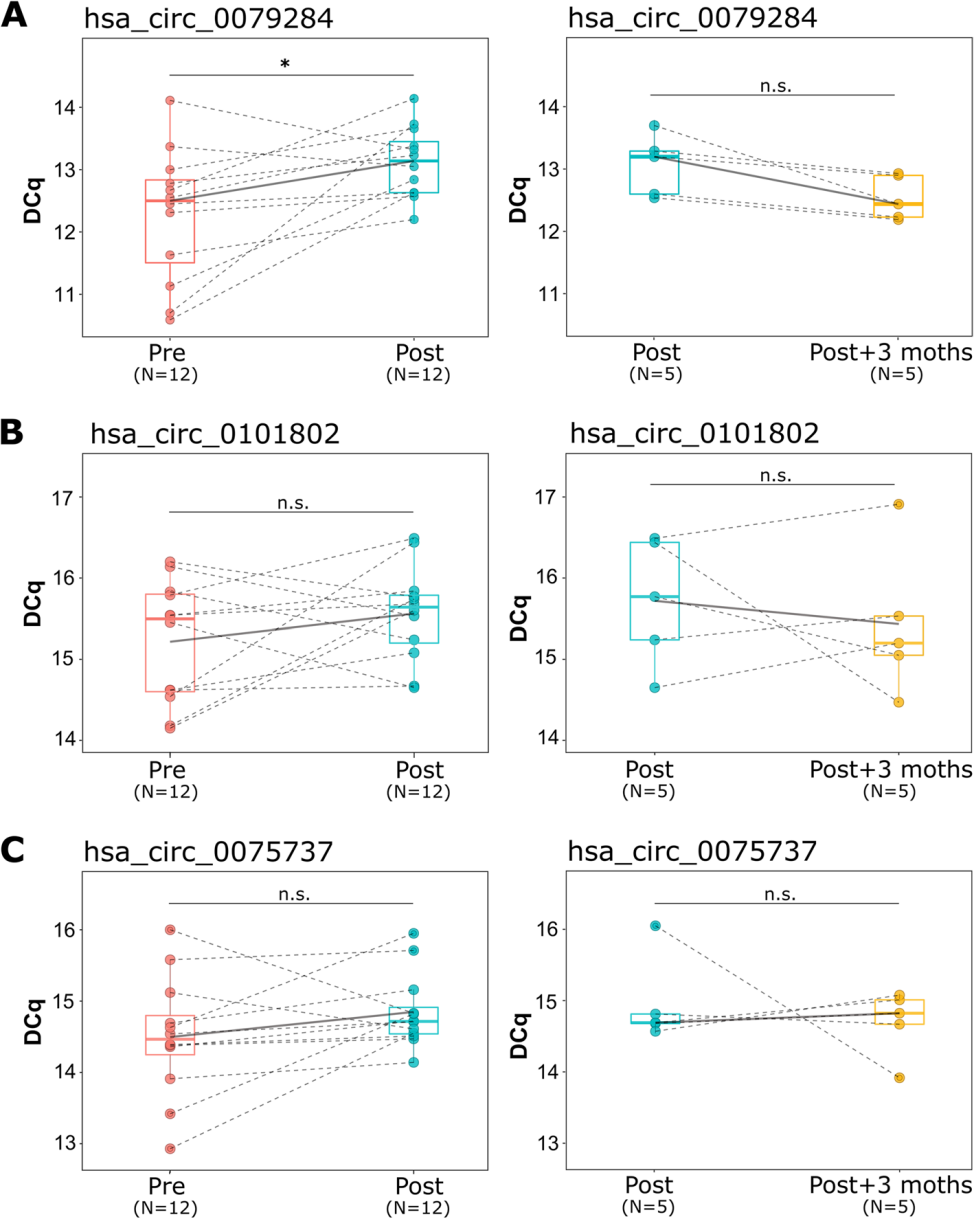
Hsa_circ_0079284 expression is reduced after physical intervention (**A**) while hsa_circ_0101802 (**B**) and hsa_circ_0075737 (**C**) are not affected. Pairwise comparisons were performed for the statistical analysis between pre intervention and post intervention samples as well as between post-intervention and 3 months after finishing the intervention. Note that a higher DCq value corresponds to a lower expression of the circRNA. Abbreviations and symbols: n.s., not significant; *, *p*-value ≤ 0.05

We found that, after the 3 months of continued exercise, hsa_circ_0079284 levels were reduced in 10 out of the 12 donors with a *p*-value of 0.02 (Fig. 5A). In the case of hsa_circ_0101802 and hsa_circ_0075737, they also show the same reduction trend after the intervention (8/12 and 10/12 donors with reduced levels respectively) although these differences were not statistically significant (Fig. 5B and C) (Supplementary Table 4). In addition, for the 5 donors from whom samples after 3 months of finishing the intervention were available, we observed a consistent (*p*-value=0.05) increase in hsa_circ_0079284 levels (Fig. 5A) (Supplementary Table 4). When the expression of hsa_circ_0079284 and TUG scores were compared right before and after the intervention, 7 out of the 12 participants obtained concordant results: reduction in hsa_circ_0079284 and TUG performance time or increased hsa_circ_0079284 and TUG performance time. Similarly, for 8 of the 12 patients, there is a concordance between the reduction in hsa_circ_0079284 levels and an increase in the SPPB score (Supplementary Fig. 3 and Supplementary Table 4).

### Insights into circRNA function: miRNA sponging and secondary structure are not predicted to be leading the way

Furthermore, we attempted to explore the biological function of the circRNAs found to be altered in frailty. The miRNA sponging function was the first described [21] and it is the most studied regulatory mechanism of circRNAs. Increasing evidence supports miRNA binding site (BS) density (number of BS/nucleotide) as a reliable criteria for assessing the miRNA sponging potential [18]. Through the prediction of TargetScan recorded in the CircInteractome database, out of the 15,189 circRNAs detected in our study, 160 circRNAs present more than 20 miRNA BS. Nevertheless, only three of those circRNAs were detected in several samples and present a miRNA BS density with at least 1 miRNA BS/100nts. (hsa_circ_0001946, hsa_circ_0106898 and hsa_circ_0106897), but none of them was found to be significantly differentially expressed with frailty.

On the other hand, recent publications have drawn attention to circRNA secondary structure as a determinant feature that can define their interaction with different proteins, and thus, their function [12, 28]. It has been reported that circRNAs can form intramolecular double-stranded regions resulting in highly structured circRNAs, which preferentially interact with proteins such as the double-stranded RNA activated protein kinase R (PKR) [28] or the UPF1 RNA binding protein and its associated protein G3BP1 [12]. Among all the 15,189 circRNAs detected in our samples, 24.7% are predicted to be highly structured (according to Fischer et al), but no differences could be observed linked to frailty status.

## Discussion

Frailty is a heterogeneous state comprising physical, psychological, and cognitive impairment, and there is no consensus on the best tool to identify frailty. Thus, new potential biomarkers that could help the identification of frailty are needed. In this line, circRNAs, which up to now have not been studied in frailty, have been shown to meet the criteria to be biomarkers. Their circular structure endows them with high resistance to RNAses, higher stability and longer half-life compared to linear RNAs [10], which eases their detection and reduces the impact of preanalytical factors during sample collection and storage. Moreover, they are evolutionary conserved and their expression has been described as tissue and developmental stage specific [31], which could increase their translatability and specificity as biomarkers. Therefore, aiming to seek for a set of circRNAs that could work as non-invasive biomarkers of frailty, in this study, we have profiled circRNA expression in frail and robust individuals by RNA-Seq.

CircRNAs have been reported to accumulate with age in *C. elegans*, as well as in *Drosophila* and mice brain aging models [7]. In humans, few studies have analyzed circRNA expression with age. Regarding non-post mitotic cells such as blood cells, the study by Wang *et al*. [39] uncovered a strong bias for circRNA upregulation during aging in human blood. Haque *et al*. [22] also performed a circRNA profile in aging human peripheral blood and found 615 circRNAs expressed in young individuals (431 young-exclusive circRNAs and 184 in common) and 1,776 circRNAs expressed in old individuals (1,592 exclusive circRNAs). These findings suggest that there is an accumulation of circRNAs with human aging. Thus, considering that all individuals enrolled in the study are older than 70 years old, a high number and concentration of circRNAs could be expected. Interestingly, in our cohort, we have detected a high variety of circRNAs (15,819 circRNAs) compared to the study by Haque et al. (1,776 circRNAs) [22] although it is worth noting that different sample preparation and bioinformatic pipelines were used. Thus, even if most of the circRNAs detected in our samples are individually quite lowly expressed, we have a mean of 20,423 circRNA-derived reads, similar to the 26,719 reads mean found by Wang et al. and considered a high abundance of circRNAs [39].

Regarding the DE circRNAs, we found a subset of 89 circRNAs that are significantly DE. Moreover, we also found a set of 4,042 frailty-exclusive circRNAs and 4,455 robust-exclusive circRNAs. Nevertheless, these subsets of group exclusive circRNAs should be taken with caution since most of them are very poorly expressed and thus, their presence or absence in each of the groups could be due to the detection limit of the technique. From the RNA-Seq analysis, 7 candidate circRNAs were selected for qPCR validation, and 4 of them were found significantly upregulated. Moreover, correlation analysis results are in line with the validated upregulation of hsa_circ_0007817, hsa_circ_0101802, hsa_circ_0060527 and hsa_circ_0075737 in frailty, since the increase in the levels of all of them is also correlated with a worsening of at least two of the three frailty scales.

Regarding the biomarker potential of these 4 differentially expressed circRNAs, ROC curves have reported very favorable individual results. Particularly, it is worth highlighting the biomarker potential of hsa_circ_0007817, whose leukocyte expression levels could be able to correctly classify frail and robust individuals with a probability of 85%. This probability increases up to 95.9% when the levels of the 3 circRNAs selected by LDA analysis are combined. Considering the high heterogeneity that characterizes the frail phenotype, these results need to be further validated in bigger cohorts, but undoubtedly set these 3 circRNAs as very promising frailty biomarkers.

We also studied the expression of these circRNAs in elderly individuals before and after a guided physical intervention. Interestingly, the expression of hsa_circ_0079248 was significantly changed after the physical intervention, which also correlated with an improvement in the functional capacity of most of the individuals based on the frailty scale scores. These findings suggest that hsa_circ_0079248 plays a role in frailty and it can inform about slight changes in functionality.

To the best of our knowledge, this study is the first to investigate the circular transcriptome in the blood of frail and robust individuals. Therefore, it is also the first to report a circRNA profile that could identify frail individuals. We have also taken a further step and, although further research will be needed, we suggest that the combination of the leukocyte expression levels of hsa_circ_0007817, hsa_circ_0075737 and hsa_circ_0079284 could be used as a biomarker of frailty.

## Materials and methods

### Participants

Samples were obtained by the Primary Care Unit of Biodonostia Health Research Institute. All participants were from the province of Gipuzkoa (Basque Country, Spain). Participants completed a questionnaire and donors with acute illness were excluded. All the methods performed in this study were carried out following the relevant guidelines. Samples were processed and stored by the Basque Biobank following their quality and legal procedures (www.biobancovasco.org).

The present study included a first cohort for the discovery and validation phases which involved individuals aged 70 or over, community-dwelling, and autonomous (Barthel > 90). The frailty status of older adults was assessed by primary care services. The incidence of frailty was evaluated by three functional tests: Timed up-and-go (TUG) [30], Gait Speed (GS) [33] and Short Physical Performance Battery (SPPB) [19]. Only the participants that were classified as frail in each of the three tests were included in the frail group. Similarly, only those individuals classified as robust in each of the three tests were included in the robust group, ensuring that the classification was consistent. The cut-off points for frailty were: TUG ≥ 12s, GS ≤ 0.8m/s, SPPB < 10 points. Participants in both groups were matched by sex and age. The main clinical and demographic characteristics of the participants are summarized in Table 1.

The second cohort included 13 elder donors that participated in a physical training program. The intervention was performed in community-based groups in the sport and leisure facilities of the municipality. Participants received 36 sessions of training including strength, flexibility, and aerobic exercises. Blood samples of participants were obtained before the start of the training and at the end of the 36 sessions (3 months). For some of them, samples were also obtained 3 months after finishing the physical intervention. At the time of collecting each of the samples, individuals were evaluated based on the TUG and SPPB frailty scales. Their main clinical and demographic data are summarized in Table 1.

### Blood sampling and RNA extraction

Whole blood samples from all the individuals enrolled in the study were collected by venipuncture in EDTA tubes (Vacutainer, BD Biosciences) and directly deposited in the Basque Biobank for subsequent processing and storage. Erythrocytes were lysed with Buffer EL (Qiagen) and total RNA from leukocytes was isolated with the miRNeasy Mini Kit (Qiagen) following the manufacturer’s instructions. RNA concentration was measured using a NanoDrop ND-1000 spectrophotometer (Thermo Scientific).

### Library preparation and RNA sequencing

For RNA sequencing, samples were pooled in groups of 3-4 individuals (3 samples per pool for males, and 4 samples per pool for females), for a total of 1.5 µg RNA per pool. Samples from donors with the same sex and frailty status were pooled. Besides, pooling was also based on frailty scale scores, pooling together the samples with similar scores. Therefore, a total of 20 pools were analyzed (10 robust and 10 frails). Library preparation and Next Generation Sequencing were performed at CD Genomics (Shirley, New York, NY, USA). The concentration and quality of the RNA samples were again measured using Bioanalyzer 2100 instrument (Agilent) before library preparation. After normalization, rRNA was depleted from the total RNA sample using the Ribo-Zero rRNA removal kit (Illumina) and followed by purification and fragmentation steps. To construct the sequencing libraries, strand-specific cDNA synthesis was performed, the 3’ ends were adenylated and adaptors were ligated. The resulting libraries were subjected to quality control and normalization process. Paired-end sequencing was performed with Illumina HiSeq X Ten (Illumina, San Diego, CA, USA) and an average of 35-50 · 10^6^ reads were obtained per sample. RNAseq data is available in the GEO repository at NCBI (GSE206762).

### CircRNA detection and quantification in RNA-Seq data

First, the quality of the sequencing was checked using FASTQC software and, then, reads were mapped to the human genome (GCHr37.p13) using STAR version 2.5.4b [8] or BWA version 0.7.17-1 [26]. Subsequently, circRNA prediction was performed by CIRCexplorer2 version 2.3.3 [42] and CIRI2 version 2.0.5 [15] adhering to the recommendation by the authors. Moreover, only circRNAs supported by both algorithms with a minimum of two reads were considered bona fide circRNAs and used in subsequent analyses, a method that has been described in the literature by other authors [20]. CircRNA expression was based on back-spliced junctions (BSJ)-spanning reads according to CIRI2 quantification. Differential expression analysis was performed using DESeq2 version 1.28.1 [29] package for R (version 3.6.3) in R-studio [35].

CircRNAs with an absolute fold-change (FC) value higher than 1.5 (FC >|1.5|) and a *p*-value less than 0.05 (*p*<0.05) were considered differentially expressed circRNAs (from now on, DE circRNAs). To select circRNAs for validation by qPCR, further filtering criteria were applied to these DE circRNAs. On one hand, circRNAs with FC >|1.8| and mean of normalized counts higher than 5 reads/sample and, on the other hand, group exclusive circRNAs, i.e. circRNAs detected in one of the groups and absent in the other, were selected.

### cDNA synthesis and Quantitative PCR

A two-step qPCR approach was carried out. Briefly, 600ng of total RNA from all individual samples were reverse transcribed into cDNA with random primers using the High Capacity cDNA Reverse Transcription Kit (Applied Biosystems, Inc., USA). The qPCR was performed in a Verity Thermal cycler (Applied Biosystems, Inc., USA) with the following program: 25ºC for 10 mins, 37ºC for 120 mins, and 85ºC for 5 mins. qPCR was performed using Power SYBRGreen Master Mix (Applied Biosystems) in a CFX384 Touch Real-Time PCR Detection System (Bio-Rad Laboratories, Inc.) following the manufacturer’s instructions. Divergent primers were designed as previously described [23] for the amplification of the circRNAs so that the amplicon spans the BSJ. B2M was used as the reference gene for normalization. Each sample was run in triplicate starting from 10ng of cDNA in 10 µl total reaction volume. qPCR cycling conditions were: 50ºC for 2 mins, 95ºC for 10 mins, 40 cycles of 95ºC for 15 sec, and 60ºC for 1 min followed by a dissociation curve analysis. The raw Cq values and melting curves were analyzed in CFX Maestro 1.0 (BioRad) where a single peak in the melting curve indicated the specificity of the amplification. The change in the expression level of the circular transcript, represented as FC, was calculated in Microsoft Excel 2010 using the 2_DDCq method.

The amplification products of all the circRNAs were subsequently purified with the ExoSAP-IT™ PCR Product Cleanup Reagent (Applied Biosystems) following the manufacturer’s instructions and Sanger sequenced (ABIprism 3130) to check for the presence of the BSJ. In addition, the PCR products were also subjected to electrophoresis on an agarose gel to confirm the presence of a single amplification product.

### Statistical analysis

CircRNA expression levels between groups were compared in R-studio using either T-test or Wilcoxon’s rank sum test, after the Shapiro-Wilcoxon normality test. The pROC package for RStudio [35] was used to generate the Receiver Operating Characteristic (ROC) curves and to calculate the area under the curve. Combined ROC curves were calculated in IBM SPSS (V 23.0). A Linear Discriminant Analysis (LDA) [14] has been performed to calculate the minimum number of variables needed to discriminate the category (Frail vs Robust). The LDA has been performed using the R-package Candisc (v0.8-6). Correlation analysis between circRNA expression and frailty scales was performed with Pearson’s or Spearman’s correlation test in R-studio for normally and non-normally distributed variables respectively. For the expression analysis in samples obtained during the physical intervention, a pairwise analysis was performed by ggwithinstats in R studio [35] between pre-intervention and post-intervention samples as well as between post-intervention and 3 months after finishing the intervention.

### Identifying circRNAs with the potential to be miRNA sponges

Dudekula et al. defined circRNAs with more than 20 miRNA binding sites (BS) as “super-sponges” for miRNAs [9]. Thus, to determine the potential of circRNAs to be miRNA sponges, BS were retrieved from CircInteractome [9] the miRNA-circRNA interactions for circRNAs with more than 20 miRNA binding sites. CircInteractome employs the TargetScan algorithm [1] to seek miRNA binding sites within circRNA sequences by surveying for 7-mer or 8-mer complementarity to the seed region as well as the 3’ end of each miRNA. Based on this dataset we assessed the number of miRNA binding sites for all the circRNAs detected by intersecting the datasets using R in RStudio.

### CircRNA structure determination

In order to predict circRNA structure, their sequence was obtained from CircInteractome. The sequence was folded *in silico* using the RNAfold function from Vienna package 2.0. to calculate the minimum thermodynamic free energy. This energy value was later normalized with the spliced length of the circRNA to calculate the length normalized minimum thermodynamic free energy (-∆G/nt) value as previously described by Fischer et al. [12]. Based on this value, circRNAs were classified as highly structured (-∆G/nt>0.25) or poorly structured (-∆G/nt<0.2).

## Supplementary Information

**Additional file 1: Supplementary Figure 1.**

**Additional file 2: Supplementary Table 1.**

**Additional file 3: Supplementary Figure 2.**

**Additional file 4: Supplementary Table 2.** Correlation analysis between circRNA expression assesed by qPCR as DCq and frailty scales in the validation cohort.

**Additional file 5: Supplementary Table 3.** Different combinations of the candidate circRNAs resulting in different ROC curves and their corresponding AUC value.

**Additional file 6: Supplementary Table 4.** Differences in circRNA expression and TUG and SPPB frailty scales’ scores between different intervention conditions for each of the participants.

**Additional file 7: Supplementary Figure 3.**

## Data Availability

The data generated in the RNAseq study is open available in Gene Expression Omnibus at the national center for Biotechnology Information (GSE206762)
