# Supplementary figures and images for: Blood RNA-Seq profiling reveals a set of circular RNAs differentially expressed in frail individuals
Source: Immun Ageing. 2023 Jul 11;20:33. doi: 10.1186/s12979-023-00356-6 (PMC10334614; doi:10.1186/s12979-023-00356-6)

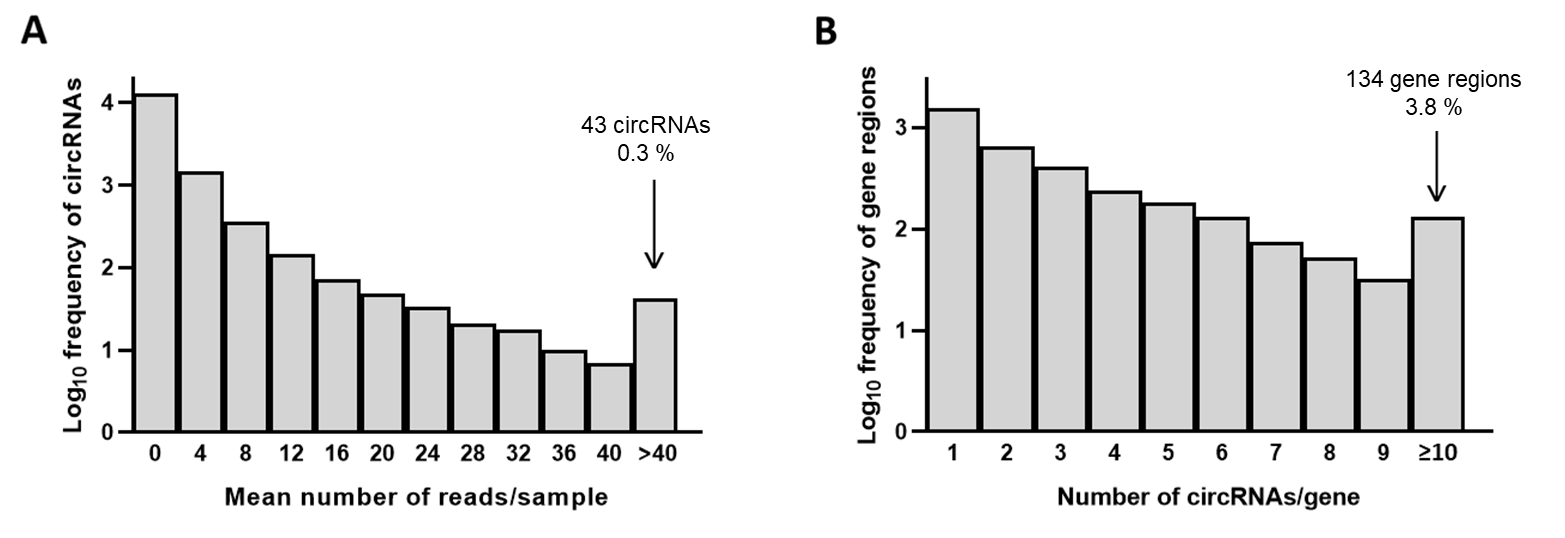

Supplement: Supplementary file 1 — Additional file 1: Supplementary Figure 1. [file 12979_2023_356_MOESM1_ESM.png]

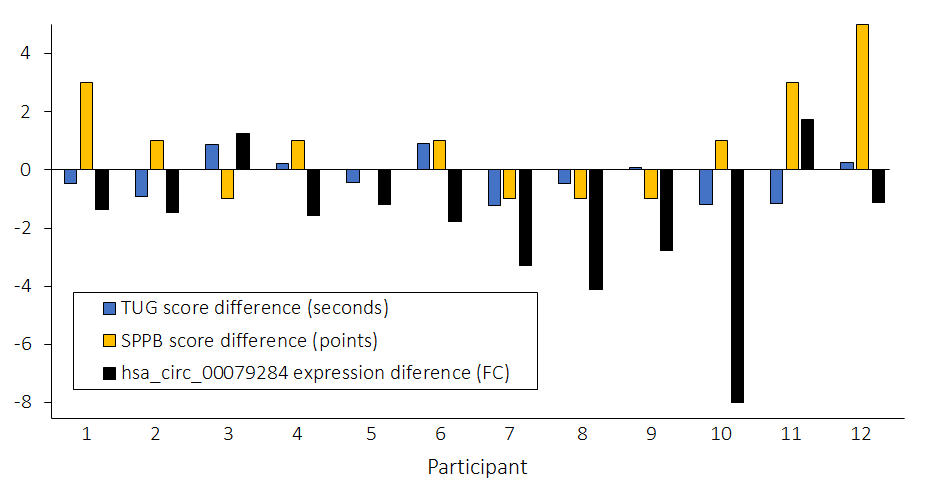

Supplement: Supplementary file 3 — Additional file 3: Supplementary Figure 2. [file 12979_2023_356_MOESM3_ESM.png]

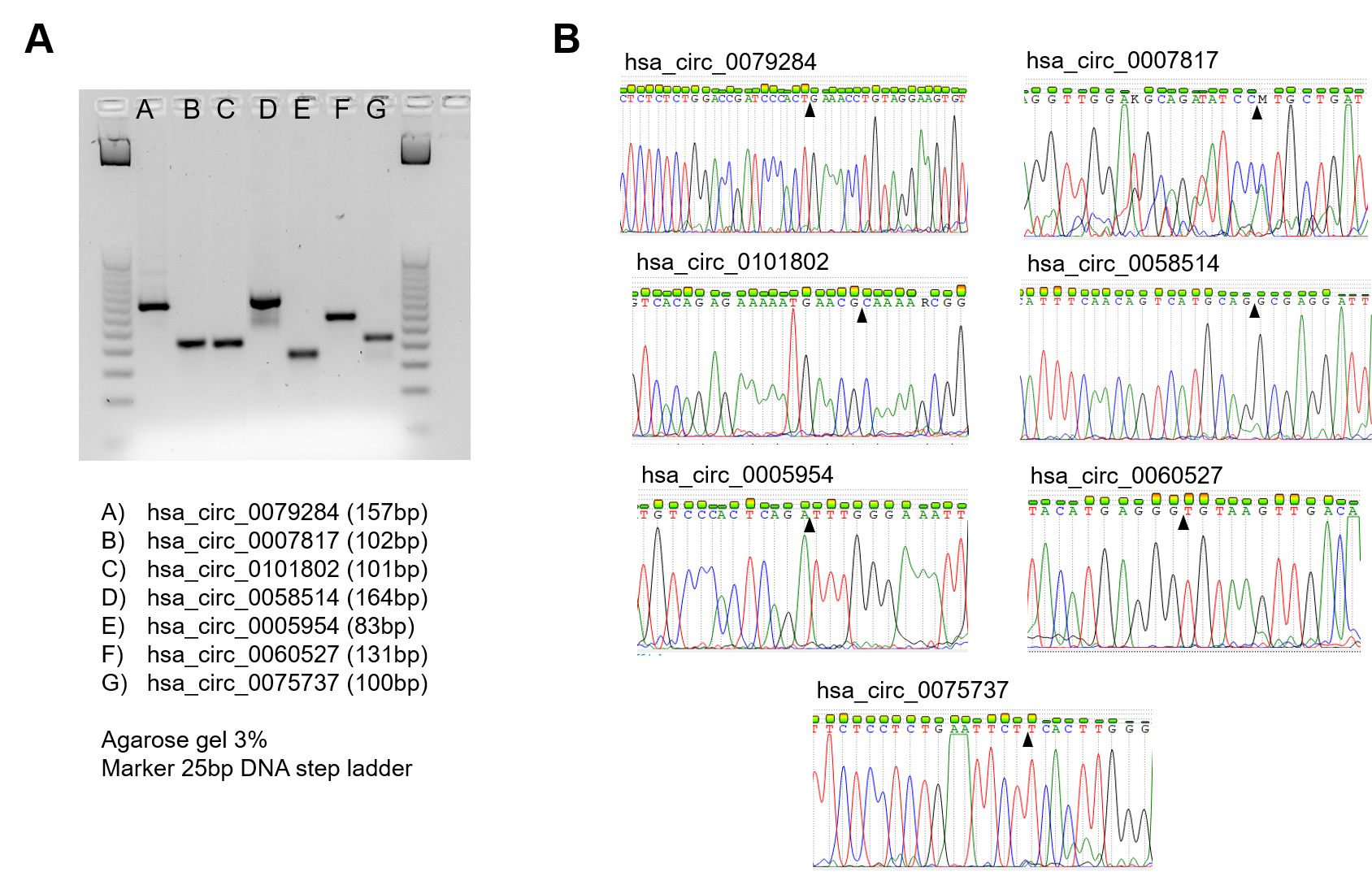

Supplement: Supplementary file 7 — Additional file 7: Supplementary Figure 3. [file 12979_2023_356_MOESM7_ESM.png]
