# Supplementary material for: Blood RNA-Seq profiling reveals a set of circular RNAs differentially expressed in frail individuals
Source: Immun Ageing. 2023 Jul 11;20:33. doi: 10.1186/s12979-023-00356-6 (PMC10334614; doi:10.1186/s12979-023-00356-6)
Supplement: Supplementary file 4 — Additional file 4: Supplementary Table 2. Correlation analysis between circRNA expression assesed by qPCR as DCq and frailty scales in the validation cohort. [file 12979_2023_356_MOESM4_ESM.docx]

| **Circbase ID** | **Genomic location (GRCh37)** | **Gene name** | **TUG**  **R (p-value)** | **GS**  **R (p-value)** | **SPPB**  **R (p-value)** |
| --- | --- | --- | --- | --- | --- |
| hsa_circ_0079284 | chr7:5680784-5692141 | RNF216 | R= -0.11 (0.37) | R= 0.069 (0.57) | R= 0.021 (0.86) |
| hsa_circ_0007817 | chrX:53641494-53642796 | HUWE1 | **R= -0.47 (<0.0001)** | **R =0.47 (<0.0001)** | **R= 0.42(0.0003)** |
| hsa_circ_0101802 | chr14:39648294-39648666 | PNN | **R= -0.29 (0.016)** | **R =0.29 (0.029)** | R=0.2 (0.094) |
| hsa_circ_0058514 | chr2:228356262-228389631 | AGFG1 | **R= -0.29 (0.016)** | R= 0.21 (0.078) | R= 0.19 (0.12) |
| hsa_circ_0005954 | chr6:111208707-111211559 | AMD1 | R= -0.22 (0.070) | R= 0.21 (0.077) | R =0.15 (0.2) |
| hsa_circ_0060527 | chr20:43610469-43615937 | STK4 | **R= -0.38 (0.0011)** | **R= 0.38 (0.0013)** | **R =0.34 (0.0045)** |
| hsa_circ_0075737 | chr6:17669523-17675264 | NUP153 | **R= -0.35 (0.0031)** | **R= 0.31 (0.0089)** | R= 0.23 (0.056) |

**Supplementary Table 2. Correlation analysis between circRNA expression assesed by qPCR as DCq and frailty scales in the validation cohort.** Pearson and Spearman correlations were performed for normaly and non-normaly distributed variables correspondingly. Note that a higher DCq value corresponds to a lower expression of the circRNA. Abbreviations: TUG, Timed up-and-go; GS, Gait Speed; SPPB, Short Physical Performance Battery
