# Supplementary material for: Blood RNA-Seq profiling reveals a set of circular RNAs differentially expressed in frail individuals
Source: Immun Ageing. 2023 Jul 11;20:33. doi: 10.1186/s12979-023-00356-6 (PMC10334614; doi:10.1186/s12979-023-00356-6)
Supplement: Supplementary file 5 — Additional file 5: Supplementary Table 3. Different combinations of the candidate circRNAs resulting in different ROC curves and their corresponding AUC value. [file 12979_2023_356_MOESM5_ESM.docx]

| **Combination** | **AUC** | **hsa_circ_0007817** | **hsa_circ_0101802** | **hsa_circ_0060527** | **hsa_circ_0075737** | **hsa_circ_0079284** |
| --- | --- | --- | --- | --- | --- | --- |
| 1 | 0.959 |  |  |  |  |  |
| 2 | 0.900 |  |  |  |  |  |
| 3 | 0.882 |  |  |  |  |  |
| 4 | 0.854 |  |  |  |  |  |
| 5 | 0.858 |  |  |  |  |  |
| 6 | 0.848 |  |  |  |  |  |
| 7 | 0.753 |  |  |  |  |  |
| 8 | 0.701 |  |  |  |  |  |
| 9 | 0.751 |  |  |  |  |  |
| 10 | 0.861 |  |  |  |  |  |
| 11 | 0.850 |  |  |  |  |  |
| 12 | 0.807 |  |  |  |  |  |

**Supplementary table 3. Different combinations of the candidate circRNAs resulting in different ROC curves and their corresponding AUC value**
