# Supplementary material for: Blood RNA-Seq profiling reveals a set of circular RNAs differentially expressed in frail individuals
Source: Immun Ageing. 2023 Jul 11;20:33. doi: 10.1186/s12979-023-00356-6 (PMC10334614; doi:10.1186/s12979-023-00356-6)
Supplement: Supplementary file 6 — Additional file 6: Supplementary Table 4. Differences in circRNA expression and TUG and SPPB frailty scales’ scores between different intervention conditions for each of the participants. [file 12979_2023_356_MOESM6_ESM.docx]

|  | **Pre-intervention vs Post-intervention** | | | | | **Post-intervention vs 3 months post-intervention** | | | | |
| --- | --- | --- | --- | --- | --- | --- | --- | --- | --- | --- |
| Participant | LAC diff (seconds) | SPPB diff (points) | Circ_0079284 FC | Circ_0101802 FC | Circ_0075737 FC | LAC diff (seconds) | SPPB diff (points) | Circ_0079284 FC | Circ_0101802 FC | Circ_0075737 FC |
| 1 | -0,46 | 3 | **-1,37** | -1,62 | -1,29 | 1,56 | -2 | 1,23 | -1,34 | -1,22 |
| 2 | -0,91 | 1 | **-1,48** | -1,03 | -1,18 | - | - | - | - | - |
| 3 | 0,86 | -1 | **1,25** | 1,52 | -1,06 | - | - | - | - | - |
| 4 | 0,23 | 1 | **-1,58** | -1,10 | -1,38 | - | - | - | - | - |
| 5 | -0,42 | 0 | **-1,20** | 1,51 | -1,12 | 1,04 | -2 | 1,27 | -1,23 | -1,10 |
| 6 | 0,91 | 1 | **-1,78** | -1,23 | -1,09 | - | - | - | - | - |
| 7 | -1,21 | -1 | **-3,27** | -2,94 | -2,65 | - | - | - | - | - |
| 8 | -0,48 | -1 | **-4,11** | -2,74 | -3,07 | - | - | - | - | - |
| 9 | 0,07 | -1 | **-2,78** | -1,38 | -1,09 | - | - | - | - | - |
| 10 | -1,18 | 1 | **-8,18** | -3,72 | -2,49 | -0,67 | -2 | 2,40 | 3,92 | 3,79 |
| 11 | -1,16 | 3 | **1,72** | 1,35 | 2,24 | 0,42 | 0 | 1,29 | 1,65 | 1,09 |
| 12 | 0,25 | 5 | **-1,13** | 1,74 | 1,42 | -0,66 | -5 | 1,29 | -1,46 | -1,37 |
| Mean diff | -0,29 | 0,92 | **-1,44** | -1,08 | -1,11 | 0,34 | -2,20 | 1,50 | 1,56 | 1,47 |
| p-value | 0,21 | 0,13 | **0,02** | 0,22 | 0,15 | 0,49 | 0,05 | 0,05 | 0,58 | 1 |

**Supplementary table 4. Differences in circRNA expression and TUG and SPPB frailty scales’ scores between different intervention conditions for each of the participants.** For frailty scales the difference in seconds or points is calculated from clinical records, the change in circRNA expression is depicted by the Fold Change (FC) assesed by qPCR
